# Differential Effects of Two Tomato Begomoviruses on the Life History and Feeding Preference of *Bemisia tabaci*

**DOI:** 10.3390/insects14110870

**Published:** 2023-11-11

**Authors:** Hsin-Yu Wu, Wei-Hua Li, Sung-Hsia Weng, Wen-Shi Tsai, Chi-Wei Tsai

**Affiliations:** 1 Department of Entomology, National Taiwan University, Taipei 106319, Taiwan; patwu@ntu.edu.tw (H.-Y.W.); f06632007@ntu.edu.tw (W.-H.L.); kgket4007@gmail.com (S.-H.W.); 2 Department of Plant Medicine, National Chiayi University, Chiayi 600335, Taiwan; wenshi.tw@mail.ncyu.edu.tw

**Keywords:** *Bemisia tabaci*, development, fecundity, longevity, preference

## Abstract

**Simple Summary:**

Tomato yellow leaf curl disease is a serious problem for tomato farmers worldwide. This disease is caused by a group of viruses, namely, tomato yellow leaf curl viruses. These viruses are mainly transmitted by the sweet potato whitefly. After the virus is ingested by the whitefly through the process of feeding on diseased plants, the virus circulates in the body of the whitefly and infects its tissues. This virus–insect relationship can influence virus transmission. The life history and feeding preference of whiteflies may be affected by viral infection in a negative, neutral, or positive manner, depending on the species of virus and whitefly involved. The tomato yellow leaf curl Thailand virus (TYLCTHV) and the tomato leaf curl Taiwan virus (ToLCTV) are common in tomato fields in Taiwan. This study examined the direct and indirect effects of TYLCTHV and ToLCTV on the life history traits (longevity, fecundity, nymph survival, and nymph developmental time) and feeding preference of whiteflies. The results revealed that TYLCTHV did not affect the life history and feeding preference of the whiteflies. However, ToLCTV infection caused low fecundity and slow development when the whiteflies fed on diseased plants. ToLCTV infection also altered the feeding preference of the whiteflies. These findings could help explain why ToLCTV is less common than TYLCTHV in tomato fields in Taiwan.

**Abstract:**

Tomato yellow leaf curl disease, caused by a group of closely related tomato yellow leaf curl viruses, is a major threat to tomato cultivation worldwide. These viruses are primarily transmitted by the sweet potato whitefly (*Bemisia tabaci*) in a persistent-circulative manner, wherein the virus circulates in the body of *B. tabaci* and infects its tissues. The complex relationship between viruses and whiteflies significantly influences virus transmission, with studies showing varying effects of the former on the life history and feeding preference of the latter. Whether these effects are direct or indirect, and whether they are negative, neutral, or positive, appears to depend on the specific interactions between virus and whitefly species. The tomato yellow leaf curl Thailand virus (TYLCTHV) and the tomato leaf curl Taiwan virus (ToLCTV) are two prevalent begomoviruses in fields in Taiwan. This study examined the direct and indirect effects of TYLCTHV and ToLCTV on the life history traits (longevity, fecundity, nymph survival, and nymph developmental time) and feeding preference of *B. tabaci* Middle East–Asia Minor 1 (MEAM1). The results revealed that TYLCTHV had no effects on these life history traits or the feeding preference of MEAM1 whiteflies. Although ToLCTV did not directly affect the longevity and fecundity of MEAM1 whiteflies, their fecundity and the nymph developmental time were negatively affected by feeding on ToLCTV-infected plants. In addition, ToLCTV infection also altered the feeding preference of MEAM1 whiteflies. The different effects of virus infection may contribute to the lower prevalence of ToLCTV compared to TYLCTHV in fields in Taiwan.

## 1. Introduction

Tomato yellow leaf curl disease, caused by a group of viruses transmitted by the sweet potato whitefly (*Bemisia tabaci* cryptic species complex), is a significant viral disease affecting tomato cultivation worldwide [1,2]. When plants become infected during early growth stages, they often fail to produce marketable fruits [3], and in severe cases, infection can result in up to 100% crop loss [4]. Furthermore, the high reproductive rate and insecticide resistance of *B. tabaci* exacerbate this problem, making this disease a limiting factor for tomato cultivation. Currently, there are no cures available for this viral disease, but there is promising research focused on enhancing tomato plant resistance to tomato yellow leaf curl viruses, as extensively reviewed by Dhaliwal et al. [5].

This disease is associated with a group of closely related viruses, i.e., tomato yellow leaf curl viruses, which belong to the genus *Begomovirus* in the family *Geminiviridae* [6]. Of these viruses, tomato yellow leaf curl virus (TYLCV)-Israel and TYLCV-Mild strains are globally distributed, spanning Africa, America, Asia, Australia/Oceania, Europe, and New Caledonia, while the other TYLCV strains (TYLCV-Kerman, TYLCV-Iran, TYLCV-Kuwait, TYLCV-Kahnooj) are prevalent throughout the Middle East [7]. These viruses are primarily transmitted by *B. tabaci* in a persistent-circulative manner, wherein the virus circulates within the body of *B. tabaci* [8]. The tropism within *B. tabaci* and successful transmission of begomoviruses by *B. tabaci* can be influenced by a multitude of factors such as *B. tabaci* cryptic species, begomovirus species, and the endosymbiont composition of *B. tabaci* [9,10,11,12,13].

Depending upon the pathosystem, numerous studies have reported that persistent viruses, including begomoviruses, can positively affect the fitness of insect vectors, either directly or indirectly through the host plant [14,15,16], whereas non-persistently transmitted viruses, which have a transient association with the insect vectors, are reported to have minimal impact on vector fitness [17]. The effects of the global pandemic pathogen TYLCV on *B. tabaci* have attracted a lot of attention. However, the outcomes of these studies regarding the effects of tomato yellow leaf curl viruses on the longevity and fecundity of *B. tabaci* cryptic species are inconsistent. Certain studies demonstrate negative effects on the longevity and fecundity of *B. tabaci* [18,19], while others suggest positive [20,21] or neutral [20,22,23,24] effects.

In contrast to direct effects, plant viruses alter the physiology of host plants, thereby affecting the insect vectors that feed on the plants. This phenomenon is referred to as an indirect effect. Plant viruses can modulate vector behavior or fitness directly or indirectly via the host plant by altering its visual appearance, the profiles of volatile organic compounds, and phytohormones [25,26,27,28]. The outcomes of indirect effects depend on the virus species, *B. tabaci* cryptic species, and host plants. There have been reports of positive [10,15,20,21,29,30,31] and negative [10,32] effects, as well as neutral effects [10,15,21,23,24].

In addition to their influence on adult whiteflies, tomato begomoviruses also directly and indirectly affect the survival and development of nymphs. The results vary depending on the specific combination of virus and *B. tabaci* cryptic species, with some studies reporting positive effects that enhance the survival of nymphs [24,31], while others report neutral effects [20,21,23,31], and there are also cases of negative effects that reduce the survival rate and prolong the developmental time [10,21,24].

Given that the effects of tomato yellow leaf curl viruses on *B. tabaci* can vary depending on the specific combination of the virus and *B. tabaci* cryptic species, this study investigated the effects of two prevalent begomoviruses in Taiwan, the tomato leaf curl Taiwan virus (ToLCTV) and the tomato yellow leaf curl Thailand virus (TYLCTHV), on the life history and feeding preference of *B. tabaci*. ToLCTV is known to be less prevalent than TYLCTHV in fields in Taiwan. The varying impact of viral infection on the whitefly may contribute to the competition among tomato begomoviruses in fields.

## 2. Materials and Methods

### 2.1. Insects, Viruses, and Plants

The sources and maintenance of *B. tabaci*, TYLCTHV, and ToLCTV were previously described [33]. We raised a colony of nonviruliferous *B. tabaci* Middle East–Asia Minor 1 (MEAM1) on Chinese kale plants (*Brassica oleracea* cv. Alboglabra Group), which are not hosts for TYLCTHV and ToLCTV. Both viruses were maintained within tomato plants (*Solanum lycopersicum* var. ANT 22) through whitefly-mediated transmission [33]. The Chinese kale and tomato plants were grown from seeds and cultured within growth chambers. The whiteflies and plants were maintained at 28 °C and 70% relative humidity (RH), with a 16:8 h (light/dark) photoperiod.

### 2.2. Effects of Virus Infection on the Longevity and Fecundity of Adults

To evaluate the direct effects of virus infection on the longevity and fecundity of *B. tabaci*, newly emerged whiteflies (0–2 days old) were infected via feeding on a virus (TYLCTHV or ToLCTV)-infected tomato plant for 24 h. After having been infected, each pair of whiteflies (one male and one female) was reared on a Chinese kale leaf enclosed within a mesh bag (6 × 15 cm, 40 mesh/cm). Separate experiments were conducted for each of the viruses. In total, 20 pairs of TYLCTHV-viruliferous whiteflies (each pair representing a replicate) were used in the experiments, and 20 pairs of nonviruliferous whiteflies served as controls. Similarly, 20 pairs of ToLCTV-viruliferous whiteflies and 20 pairs of nonviruliferous whiteflies were used in the experiments. The survival of the whiteflies was recorded daily until all the test whiteflies died. We transferred the whiteflies to another Chinese kale leaf every 7 days to collect eggs laid within a week. Hatched and unhatched eggs on the former leaves were counted under a dissecting microscope to calculate the fecundity. Each experiment was repeated thrice.

To evaluate the indirect effects of virus infection on the longevity and fecundity of *B. tabaci*, each pair (one male and one female) of newly emerged whiteflies (0–2 days old) was reared on a virus (TYLCTHV or ToLCTV)-infected tomato leaf enclosed within a mesh bag (6 × 15 cm, 40 mesh/cm). Separate experiments were conducted for each of the viruses. In total, 20 pairs of whiteflies reared on TYLCTHV-infected tomato leaves (each pair representing a replicate) were used in the experiments, and 20 pairs of nonviruliferous whiteflies reared on healthy tomato leaves served as controls. Similarly, 20 pairs of whiteflies reared on ToLCTV-infected tomato leaves and 20 pairs of nonviruliferous whiteflies reared on healthy tomato leaves were used in the experiments. Then, the survival of the whiteflies and the number of hatched and unhatched eggs were recorded as described above. Each experiment was repeated thrice.

### 2.3. Effects of Virus Infection on the Survival and Development of Nymphs

Because first-instar nymphs of *B. tabaci* crawl to find suitable feeding sites and begin with a sedentary lifestyle, forcibly transferring them to another plant would result in mortality. Therefore, whitefly nymphs were examined only on tomato leaves. Separate experiments were conducted for each of the viruses. Nonviruliferous adult females were individually reared on a virus (TYLCTHV or ToLCTV)-infected tomato leaf enclosed in a mesh bag as described above. In total, 10 females reared on TYLCTHV-infected leaves and 13 females reared on ToLCTV-infected leaves were used in the experiment (each individual female whitefly reared on a virus-infected leaf representing a replicate), while 10 and 13 nonviruliferous adult females individually reared on a healthy tomato leaf served as controls. After a 72 h oviposition period, the females were removed with a mouth aspirator, and the number of eggs on each leaf was recorded. Because *B. tabaci* takes approximately 3 weeks to develop from eggs to adults on tomato leaves at 28 °C, starting from the 14th day after the removal of the females, the number of newly emerged adults was recorded daily. The survival rate of nymphs and the developmental periods from eggs to adults were calculated based on the aforementioned data. We assumed that time zero for calculating the developmental period was the end of the 72 h oviposition period. We acknowledge that, as a result, the development time might be slightly underestimated.

### 2.4. Effects of Virus Infection on the Feeding Preference of Adults

To examine the effects of virus infection on feeding preference, one virus (TYLCTHV or ToLCTV)-infected and one healthy tomato plant were placed in a net cage (47.5 × 47.5 × 47.5 cm, Megaview, Taichung, Taiwan) for the whiteflies to choose from. A total of 40 viruliferous or 40 nonviruliferous whiteflies were collected and enclosed in a 1.5 mL microcentrifuge tube and then released from the center of the distance between two test plants. Then, the number of whiteflies that settled on each plant was recorded at 3, 6, 9, 12, and 24 h after the whiteflies were released. Each experiment was replicated 10 times.

### 2.5. Statistical Analysis

The longevity, fecundity, nymph survival, and developmental period data were analyzed using Student’s *t* test when the data were normally distributed. Non-normally distributed data were analyzed using the Mann–Whitney test. Repeated-measure ANOVA was used to evaluate the effects of virus infection on feeding preference when the data were normally distributed; otherwise, mixed-model analysis was used.

## 3. Results

### 3.1. Direct Effects of Virus Infection on the Longevity of Adults

The average longevities of the viruliferous and nonviruliferous adults are listed in Table 1. There was no significant difference between the longevities of TYLCTHV-viruliferous and nonviruliferous whiteflies (Mann–Whitney test, males: *p* = 0.17; females *p* = 0.60). Similarly, there was no significant difference between the longevities of ToLCTV-viruliferous and nonviruliferous whiteflies (Mann–Whitney test, males: *p* = 0.63; females: *p* = 0.75). These results collectively indicate that neither TYLCTHV nor ToLCTV had direct effects on the longevity of adult *B. tabaci*.

### 3.2. Direct Effects of Virus Infection on the Fecundity of Adults

The average number of eggs of viruliferous and nonviruliferous adult females is listed in Table 2. There was no significant difference between the lifelong egg productions of TYLCTHV-viruliferous and nonviruliferous whiteflies (Student’s *t* test, *p* = 0.76). When lifelong egg production was divided by the number of days of oviposition, the daily egg production was acquired, and there was no significant difference between the daily egg production of TYLCTHV-viruliferous and nonviruliferous whiteflies (Student’s *t* test, *p* = 0.29). Similarly, there was no significant difference between the numbers of eggs of ToLCTV-viruliferous and nonviruliferous whiteflies (Student’s *t* test, lifelong egg production: *p* = 0.37; daily egg production: *p* = 0.30). These results collectively indicate that neither TYLCTHV nor ToLCTV had direct effects on the fecundity of *B. tabaci*. 

### 3.3. Indirect and Direct Effects of Virus Infection on the Longevity of Adults

While the intent was to assess the indirect effects of virus infection on the longevity of whiteflies by comparing the nonviruliferous whiteflies that fed on healthy plants to those that fed on virus-infected plants, previous research has shown that nonviruliferous whiteflies that fed on virus-infected plants become viruliferous within 3–14 h [24]. The rapid transition to a viruliferous state meant that the comparison would ultimately be between nonviruliferous whiteflies that fed on healthy plants and viruliferous whiteflies that fed on virus-infected plants. As a result, both indirect and direct effects were involved in this experiment. The longevity of adult whiteflies displayed no significant difference whether they fed on healthy or TYLCTHV-infected plants (Table 3, Mann–Whitney test, males: *p* = 0.45; females: *p* = 0.27). Similarly, the longevity of adult whiteflies displayed no significant difference whether they fed on healthy or ToLCTV-infected plants (Table 3, Mann–Whitney test, males: *p* = 0.97; females: *p* = 0.37). These results suggest that neither TYLCTHV nor ToLCTV had direct or indirect effects on the longevity of adult *B. tabaci*.

### 3.4. Indirect and Direct Effects of Virus Infection on the Fecundity of Adults

For the same reason described above, specifically, that nonviruliferous whiteflies can rapidly become viruliferous, both indirect and direct effects were involved in the experiment comparing the fecundities of nonviruliferous whiteflies that fed on healthy plants and viruliferous whiteflies that fed on virus-infected plants. No significant difference in lifelong or daily egg production was observed between adult females that fed on healthy or TYLCTHV-infected plants (Table 4, Mann–Whitney test, lifelong egg production: *p* = 0.74; daily egg production: *p* = 0.18). On the contrary, adult females that fed on ToLCTV-infected plants laid fewer eggs than those that fed on healthy plants (Table 4, Mann–Whitney test, lifelong egg production: *p* < 0.001; daily egg production: *p* < 0.001). These results suggest that ToLCTV infection, but not TYLCTHV infection, indirectly affected the fecundity of *B. tabaci*.

### 3.5. Indirect and Direct Effects of Virus Infection on the Survival and Development of Nymphs

The average survival rate and developmental time of *B. tabaci* nymphs that fed on healthy or virus-infected plants are listed in Table 5. There was no significant difference between the survival rates of nymphs that fed on healthy or TYLCTHV-infected plants (Student’s *t* test, *p* = 0.55). Similarly, the survival rates of nymphs that fed on healthy or ToLCTV-infected plants were not significantly different (Student’s *t* test, *p* = 0.86). The development time of nymphs that fed on healthy plants was also not significantly different compared to nymphs that fed on TYLCTHV-infected plants (Student’s *t* test, *p* = 0.57). In contrast, the nymphal stage of whiteflies that fed on ToLCTV-infected plants was longer than that of those that fed on healthy plants (Student’s *t* test, *p* < 0.001). These results suggest that ToLCTV infection, but not TYLCTHV infection, affected the developmental time of *B. tabaci* nymphs.

### 3.6. Effects of Virus Infection on the Feeding Preference of Adults

**Figure 1:**
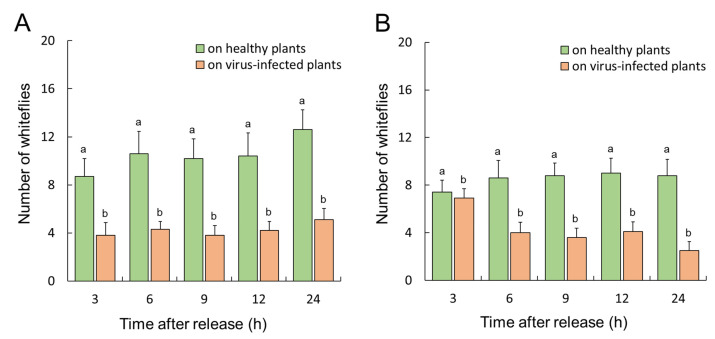
Feeding preferences of adult *B. tabaci* for tomato plants. Nonviruliferous whiteflies (**A**) or TYLCTHV-viruliferous whiteflies (**B**) that fed on healthy plants (green columns) or TYLCTHV-infected plants (orange columns). Different lowercase letters indicate significant differences (**A**), repeated-measure ANOVA, *p* < 0.05; (**B**), mixed-model analysis, *p* < 0.05. Error bars represent the standard error of the mean.

**Figure 2:**
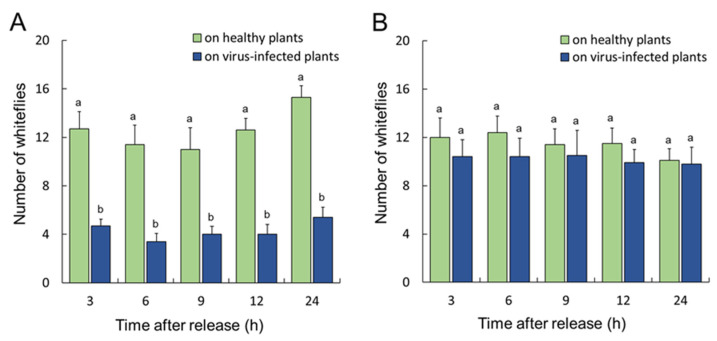
Feeding preferences of adult *B. tabaci* for tomato plants. Nonviruliferous whiteflies (**A**) or ToLCTV-viruliferous whiteflies (**B**) that fed on healthy plants (green columns) or ToLCTV-infected plants (blue columns). Different lowercase letters indicate significant differences (**A**), mixed-model analysis, *p* < 0.05; (**B**), repeated-measure ANOVA, *p* < 0.05). Error bars represent the standard error of the mean.

Nonviruliferous whiteflies preferred to feed on healthy plants rather than on TYLCTHV-infected plants (Figure 1A, repeated-measure ANOVA, *p* = 0.004). TYLCTHV-viruliferous whiteflies exhibited a feeding preference for healthy plants over TYLCTHV-infected plants (Figure 1B, mixed-model, *p* = 0.004). Similarly, nonviruliferous whiteflies preferred to feed on healthy plants compared to ToLCTV-infected plants (Figure 2A, mixed-model, *p* < 0.001). In contrast, ToLCTV-viruliferous whiteflies did not exhibit a feeding preference between healthy plants and ToLCTV-infected plants (Figure 2B, repeated-measure ANOVA, *p* = 0.35). The results demonstrate that *B. tabaci* exhibited a feeding preference for healthy plants, whereas ToLCTV infection altered its feeding preference.

## 4. Discussion

Plant viruses can induce various effects on insect vectors by causing alterations in the physiology of the insect vector [17,29,34,35]. TYLCTHV and ToLCTV are transmitted by *B. tabaci* in a persistent-circulative manner [33,36], and as begomoviruses infect various organs and tissues of whitefly, they have the potential to exert direct effects on their vectors. Our results indicate that TYLCTHV and ToLCTV infections did not directly affect the longevity and fecundity of *B. tabaci* MEAM1. Similarly, the majority of studies that have investigated TYLCV have reported neutral effects on the longevity and fecundity of *B. tabaci* Mediterranean (MED) [22,23,24]. However, for MEAM1 whiteflies, the direct effects of tomato begomovirus infection have been reported to be negative [20,24], positive [37], or neutral [38].

Plant viruses may also affect insect vectors indirectly by altering the physiological properties of host plants, which include changing the nutritive value, suppressing plant defenses, or modifying the secondary plant compounds [29,39,40,41,42,43]. Our results indicate that TYLCTHV infection did not indirectly affect the longevity and fecundity of *B. tabaci* MEAM1, but ToLCTV infection indirectly affected fecundity. Similarly, most previous studies have reported indirect neutral effects of TYLCV on the longevity of MEAM1, MED, and Asia II 1 whiteflies [10,23,24], whereas studies on tomato yellow leaf curl China virus (TYLCCNV) have revealed indirect positive effects on their longevity [10,20,21,31]. Most studies suggest that TYLCV and TYLCCNV have positive indirect effects on the fecundity of MEAM1, MED, and Asia I whiteflies [15,20,21,24,30,31,41] or neutral indirect effects on MEAM1, Asia II 1, and Asia II 3 whiteflies [10,15,20,21,23,24]. Only two studies have shown negative indirect effects of TYLCV and TYLCCNV on the fecundity of MEAM1 and Asia II 3 whiteflies [8,22].

Mauck et al. [34,44] conducted a meta-analysis and concluded that, in most cases, persistent plant viruses like begomoviruses tend to enhance vector fitness (reduced developmental time or increased fecundity and survival) by either improving plant nutrient content or by suppressing plant defenses. For instance, TYLCCNV infection in plants leads to the suppression of terpenoid synthesis and jasmonic-acid-mediated defenses, resulting in the enhanced fitness of MEAM1 whiteflies [41,45]. These suppressions are attributed to a pathogenicity factor encoded within the betasatellite [43,45]. However, recent studies including the current study point towards pathosystem-specific whitefly–begomovirus interactions. In the current study, two tomato begomoviruses interacted with the same population of MEAM1 whiteflies differentially under similar experimental conditions. Similarly, studies conducted using the same population of MEAM1 whiteflies under similar conditions and using two different begomoviruses, TYLCV and cucurbit leaf crumple virus (CuLCrV), have reported that TYLCV infection in susceptible cultivars provided fitness benefits to MEAM1 whiteflies, while CuLCrV infection in susceptible plants had no effect on vector fitness [12,46]. Furthermore, infection with a particular begomovirus in different plants can lead to differential interactions between the host plant and vectors, with profound implications for virus transmission and epidemics. For instance, sida golden mosaic virus (SiGMV) in different host plants resulted in negative, neutral, and positive effects on the fitness of MEAM1 whiteflies [47]. Taken together, the results from previous and current studies suggest that rather than generalizing the begomovirus–host–vector interactions, localized pathosystem-specific studies are warranted to comprehend the interactions within pathosystems in order to develop ecologically and economically sound whitefly–virus management programs.

In addition to the effects of virus infection on adult vectors, viruses may also affect the survival and development of nymphs. In this study, feeding on TYLCTHV- or ToLCTV-infected plants did not affect the survival rate of MEAM1 nymphs; however, the developmental time increased when the nymphs fed on ToLCTV-infected plants. Nevertheless, there is still no consensus on the effects of tomato begomoviruses on whitefly nymphs. Some studies reported that TYLCV did not affect the survival and development of the nymphs of various species [23,31], while negative [24] and positive [24,31,46] effects have also been reported. Furthermore, TYLCCNV has been reported to negatively affect the survival and development of MED and Asia II 1 nymphs [21]. Based on the results of these studies, the effects of tomato begomoviruses on *B. tabaci* nymphs may vary depending on the specific combination of virus and *B. tabaci* cryptic species.

In this study, nonviruliferous MEAM1 whiteflies preferred to feed on healthy plants. A feeding preference for healthy plants has been reported for MEAM1 whiteflies, but not for MED whiteflies [31,48,49]. Therefore, the feeding preference of *B. tabaci* may be species-specific. MEAM1 whiteflies infected with ToLCTV, but not TYLCTHV, showed no feeding preference. Similar to the case of TYLCTHV, TYLCV-viruliferous MEAM1 whiteflies have previously been shown to have no feeding preference between healthy and virus-infected plants [48]. Plants infected with persistent viruses are hypothesized to attract insect vectors for feeding, thereby increasing the chance of virus transmission [17,34,35]. However, this hypothesis is true only for MED whiteflies that prefer TYLCV-infected plants.

In this study, we reported that ToLCTV interacted negatively with the vector, in contrast to TYLCTHV. Previous studies demonstrated that closely related begomoviruses can interact differently with different *B. tabaci* cryptic species, resulting in variations in virus accumulation and transmission [12,50,51]. Furthermore, even within TYLCV strains, a few amino acid differences can lead to distinct interactions in the vector’s body, affecting transmission [52,53]. Since TYLCTHV and ToLCTV share a common host and vector, it is plausible that they may interact with each other within the vector. However, the consequences of a mixed infection of TYLCTHV and ToLCTV on host–vector interactions and transmission remain unclear at this time. Additionally, we do not yet understand how both viruses will interact with *B. tabaci* Asia II 6 (formerly the Nauru biotype) and Australia (formerly the An biotype), which have previously been documented in Taiwan [54]. Both these venues warrant further investigations.

## 5. Conclusions

TYLCTHV had neither direct nor indirect effects on the longevity, fecundity, nymph survival, nymph development, and feeding preference of *B. tabaci* MEAM1. In contrast, ToLCTV did not directly affect the longevity and fecundity of MEAM1 whiteflies but had negative indirect effects on fecundity and nymph development. In addition, ToLCTV infection altered the feeding preference of MEAM1 whiteflies. The negative effects of ToLCTV infection on the fecundity and nymph development hindered the increase in the size of ToLCTV-viruliferous populations. Unlike TYLCTHV-viruliferous whiteflies that preferred to feed on healthy plants, ToLCTV-viruliferous whiteflies did not have a feeding preference for healthy plants, reducing the probability of virus transmission. These different results may contribute to the lower prevalence of ToLCTV than TYLCTHV in fields in Taiwan.

## Figures and Tables

**Table 1:** Direct effects of virus infection on the longevity of adult *B. tabaci*.

Virus	Sex	Longevity (Mean ± SEM, Days)				
		*n*	Viruliferous Whiteflies	*n*	Nonviruliferous Whiteflies	*p*-Value ^3^
TYLCTHV ^1^	Male	43	24.4 ± 1.9	45	21.1 ± 1.9	0.17
	Female	45	39.2 ± 2.7	48	41.4 ± 2.5	0.60
ToLCTV ^2^	Male	44	21.4 ± 1.8	56	22.6 ± 1.7	0.63
	Female	52	42.2 ± 2.3	55	41.3 ± 2.3	0.75

^1^ TYLCTHV, tomato yellow leaf curl Thailand virus; ^2^ ToLCTV, tomato leaf curl Taiwan virus; ^3^ Pairwise comparisons were conducted within virus treatments using the Mann–Whitney test.

**Table 2:** Direct effects of virus infection on the fecundity of female *B. tabaci*.

Virus	Whiteflies	*n*	Number of Eggs/Female ^1^ (Mean ± SEM)	Number of Eggs/Female/Day ^1^ (Mean ± SEM)
TYLCTHV	Viruliferous	44	233.4 ± 19.1 a	6.3 ± 0.4 a
	Nonviruliferous	48	241.6 ± 18.2 a	5.8 ± 0.3 a
ToLCTV	Viruliferous	50	258.0 ± 19.4 a	6.7 ± 0.4 a
	Nonviruliferous	55	235.4 ± 16.4 a	6.3 ± 0.3 a

^1^ Same lowercase letters indicate no significant differences between viruliferous and nonviruliferous within virus treatments (Student’s *t* test, *p* > 0.05).

**Table 3:** Indirect and direct effects of virus infection on the longevity of adult *B. tabaci*.

Virus	Sex	Longevity (Mean ± SEM, Days)				
		*n*	on Virus-Infected Plants	*n*	on Healthy Plants	*p*-Value ^1^
TYLCTHV	Male	47	14.3 ± 1.2	53	15.1 ± 1.0	0.45
	Female	48	23.3 ± 1.2	51	21.8 ± 1.5	0.27
ToLCTV	Male	46	12.8 ± 1.2	43	11.6 ± 0.7	0.97
	Female	41	16.7 ± 1.6	46	16.2 ± 0.8	0.37

^1^ Pairwise comparisons were conducted within virus treatments using the Mann–Whitney test.

**Table 4:** Indirect and direct effects of virus infection on the fecundity of female *B. tabaci*.

Virus	Status of Plants	*n*	Number of Eggs/Female ^1^ (Mean ± SEM)	Number of Eggs/Female/Day ^1^ (Mean ± SEM)
TYLCTHV	Virus-infected	46	37.3 ± 3.8 a	1.9 ± 0.2 a
	Healthy	48	44.6 ± 5.9 a	2.5 ± 0.2 a
ToLCTV	Virus-infected	40	23.0 ± 3.3 a	1.5 ± 0.2 a
	Healthy	46	38.2 ± 3.3 b	3.1 ± 0.3 b

^1^ Different lowercase letters indicate significant differences between whiteflies feeding on virus-infected plants and healthy plants within virus treatments (Mann–Whitney test, *p* < 0.05).

**Table 5:** Indirect and direct effects of virus infection on the survival and development of *B. tabaci* nymphs.

Virus	Status of Plants	*n*	Survival Rate (%) ^1^ (Mean ± SEM)	*n*	Developmental Time (Days) ^1^ (Mean ± SEM)
TYLCTHV	Virus-infected	10	43.2 ± 12.9 a	28	21.6 ± 0.8 a
	Healthy	10	32.8 ± 10.8 a	21	21.1 ± 0.4 a
ToLCTV	Virus-infected	13	26.8 ± 9.6 a	34	25.2 ± 0.6 a
	Healthy	13	29.1 ± 8.4 a	47	20.9 ± 0.4 b

^1^ Different lowercase letters indicate significant differences between whiteflies feeding on virus-infected plants and healthy plants within virus treatments (Student’s *t* test, *p* < 0.05).

## Data Availability

The data presented in this study are available on request from the corresponding author.
